# Complete genome sequence of *Bradyrhizobium* NP1, isolated from forest soil

**DOI:** 10.1128/MRA.00585-23

**Published:** 2023-09-21

**Authors:** Trevor Fisher, Francesca Durmazolu, Kristen M. DeAngelis, Maureen A. Morrow

**Affiliations:** 1 Department of Biology, State University of New York at New Paltz, New Paltz, New York, USA; 2 Department of Microbiology, University of Massachusetts, Amherst, Massachusetts, USA; Wellesley College, Wellesley, Massachusetts, USA

**Keywords:** *Bradyrhizobium*, forest soil

## Abstract

We report the complete genome sequence of *Bradyrhizobium* strain NP1. This bacterium was isolated from forest soil that had been subject to chronic warming. The genome of this novel isolated bacteria is presented as a single circular contig of 7,712,921 base pairs with 64.14% GC content.

The diverse genus *Bradyrhizobium* is predicted to contain approximately 800 species (1) and includes non-symbiotic species that dominate forest soil (2). *Bradyrhizobium* NP1 was isolated from the Prospect Hill long-term warming experiment (3) at the Harvard Forest Long-Term Ecological Research site in Petersham, MA (42.54–72.18). In May 2021, organic horizon soil was collected from a heated plot and stored at 4°C until use. In August 2021, soil was plated on dilute nutrient broth supplemented with ammonium nitrate (Table 1) and incubated at room temperature (22°C). NP1 was a slow-growing colony that appeared after 10 weeks and thus was chosen for analysis.

**TABLE 1:** Isolation medium

Ingredient	Amount per liter
Difco^TM^ nutrient broth	0.08 g
NH_4_NO_3_	0.5 g
1 M CaCl	0.6 mL
Agar	6.0 g
Gellan gum	6.4 g
Cyclohexamide	50 mg

NP1 was identified as a *Bradyrihzobuim* using the online NCBI BLASTn tool (standard database) with a 16S rRNA gene sequence amplified by PCR (universal primers 27F and 1492R) from genomic DNA extracted with the Quick-DNA Fecal/Soil Microbe Miniprep Kit (Zymo, Irvine, CA) (GenBank accession number OR045828). The sequence has 99.6% identity with at least 100 species of *Bradyrhizobium*.

NP1 was streaked to purity and grown in 10% Tryptic Soy broth (Becton, Dickinson and Company, Sparks, MD) with 1X MEM Vitamin Solution (Gibco, Grand Island, NY) at 30°C for 2 days with shaking. Genomic DNA was extracted with the DNeasy Blood and Tissue Kit using a 1-hour lysozyme pre-treatment and the RNase A step. (Qiagen, Hilden, Germany). The DNA was quantified using a Qubit 4.0 fluorometer (ds DNA HS Assay; Invitrogen, Waltham, MA). Whole-genome sequencing (WGS) was performed using the Illumina DNA Prep Kit and IDT 10 bp UDI indices on an Illumina NextSeq 2000 (2 × 151 bp reads) by SeqCenter (Pittsburgh, PA). Demultiplexing, quality control, and adapter trimming were performed with the proprietary bcl-convert (v3.9.30), resulting in 7,223,840 reads. The reads were trimmed with Trimmomatic (v0.36) (4) in the DOE Systems Biology Knowledgebase (KBase) platform (5) using default parameters. The resultant 7,115,010 reads had an average read length of 145.82  ±  17.45 (134× coverage).

The same DNA sample was sequenced at Plasmidsaurus (Eugene, OR). The library was constructed with the Oxford Nanopore Technologies Ligation Sequencing Kit version SQK-LSK115 (no shearing or size selection) and was sequenced on GridION 10.4.1 flowcells (FLO-MIN114) using the “Super accuracy” basecaller in MinKNOW. The reads were filtered with Filtlong (v.0.2.10; https://github.com/rrwick/Filtlong) in KBase (5) to remove reads <1,000 nucleotides and 5% of the lowest quality. A total of 52,003 reads were obtained (average read length, 8030.49  ±  6726.47, 54× coverage).

A hybrid assembly was generated with Unicyclyer(v0.4.8) (6) in KBase, with rotation between multiple rounds of polishing, resulting in a single circular contig of 7,712,921 base pairs (64.14% GC, N_50_ = 7,712,921). CheckM (v1.0.18, KBase) (7) showed 99.98% completeness and 1.01% estimated contamination. The Prokaryotic Genome Annotation Pipeline annotation (8) predicted 7,428 genes, 7,277 proteins, 48 tRNAs, and 3 rRNAs. Taxonomy analysis of the RASTtk(v1.073) (9) annotated genome in KBase using GTDB-Tk(v1.7.0)(10) produced a match with *Bradyrhizobium* but fell outside the ANI radius for a closest species match.

Complete pathways for nodulation and nitrogen fixation are not present, suggesting that NP1 is free-living. The availability of this genome will contribute to our understanding of this genus.
